# Lipid dysfunction and pathogenesis of multiple system atrophy

**DOI:** 10.1186/2051-5960-2-15

**Published:** 2014-02-07

**Authors:** Jonathan M Bleasel, Joanna H Wong, Glenda M Halliday, Woojin Scott Kim

**Affiliations:** 1 Neuroscience Research Australia, Barker St, Randwick, Sydney NSW 2031, Australia; 2 School of Medical Sciences, University of New South Wales, Sydney NSW 2052, Australia

**Keywords:** Multiple system atrophy, Lipid, α-synuclein, Oligodendrocyte, COQ2, ABCA8

## Abstract

Multiple system atrophy (MSA) is a progressive neurodegenerative disease characterized by the accumulation of α-synuclein protein in the cytoplasm of oligodendrocytes, the myelin-producing support cells of the central nervous system (CNS). The brain is the most lipid-rich organ in the body and disordered metabolism of various lipid constituents is increasingly recognized as an important factor in the pathogenesis of several neurodegenerative diseases. α-Synuclein is a 17 kDa protein with a close association to lipid membranes and biosynthetic processes in the CNS, yet its precise function is a matter of speculation, particularly in oligodendrocytes. α-Synuclein aggregation in neurons is a well-characterized feature of Parkinson’s disease and dementia with Lewy bodies. Epidemiological evidence and *in vitro* studies of α-synuclein molecular dynamics suggest that disordered lipid homeostasis may play a role in the pathogenesis of α-synuclein aggregation. However, MSA is distinct from other α-synucleinopathies in a number of respects, not least the disparate cellular focus of α-synuclein pathology. The recent identification of causal mutations and polymorphisms in *COQ2*, a gene encoding a biosynthetic enzyme for the production of the lipid-soluble electron carrier coenzyme Q_10_ (ubiquinone), puts membrane transporters as central to MSA pathogenesis, although how such transporters are involved in the early myelin degeneration observed in MSA remains unclear. The purpose of this review is to bring together available evidence to explore the potential role of membrane transporters and lipid dyshomeostasis in the pathogenesis of α-synuclein aggregation in MSA. We hypothesize that dysregulation of the specialized lipid metabolism involved in myelin synthesis and maintenance by oligodendrocytes underlies the unique neuropathology of MSA.

## Introduction

Multiple system atrophy (MSA) is a progressive neurodegenerative disease characterized by the clinical triad of parkinsonism, cerebellar ataxia and autonomic failure. The distribution of pathology classically encompasses three functional systems in the central nervous system (CNS); the striatonigral system, olivopontocerebellar system and autonomic nuclei of the brainstem and spinal cord [1,2]. MSA typically affects individuals in the sixth decade of life or later [3] with a mean survival of approximately 9 years [4,5]. The annual incidence in the age group 50–99 years has been estimated at 3 cases per 100,000 person years [6,7]. While the general consensus is that MSA is a highly sporadic disease, emerging evidence has suggested rare genetic variants increase susceptibility, although this appears to be dependent on the geographical distribution of sample patients [8-11]. Furthermore, albeit scant, evidence suggesting environmental risk factors similarly increasing susceptibility has also been reported [12-16].

**Figure 1:**
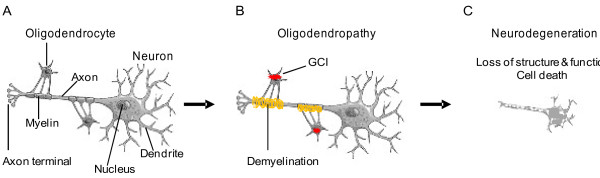
**A putative model of oligodendrocyte dysfunction in multiple system atrophy. (A)** Normal oligodendrocytes with myelin covering the axon of a neuron. **(B)** Formation of glial cytoplasmic inclusions (GCIs), composed of aggregated α-synuclein, and demyelination. **(C)** Demyelination is followed by neurodegeneration and cell death.

The pathological hallmark of MSA is the appearance of glial cytoplasmic inclusions (GCIs) in oligodendrocytes (Figure 1), the myelin producing support cells of the central nervous system (CNS). GCIs comprise insoluble proteinaceous filaments composed chiefly of α-synuclein (α-syn) [17]. α-Syn is a 17-kDa protein encoded by the *SNCA* gene at the cytogenetic location 4q22.1. It is predominantly expressed in neurons where it appears to localize in the synaptic terminal and play a role in vesicle transport and turnover [18-20]. Deposits of insoluble α-syn also occur in neurons in Parkinson’s disease (PD) and dementia with Lewy bodies (DLB), although the filament size and morphology in these inclusions differ somewhat from that of GCIs [21]. The causes of α-syn aggregation and the reason for the unique oligodendrocyte focus of pathology in MSA remain poorly understood. However, in line with advances in understanding of other neurodegenerative proteinopathies, including Alzheimer’s disease [22,23], the metabolism of lipids in the brain is emerging as an important focus in the study of proteinopathies and α-syn pathophysiology.

For α-syn, such lines of evidence stem from studies which demonstrate that α-syn readily binds to lipid droplets and phospholipid bilayers *in vitro*, whereby lipid membrane association induces folding of the protein from its unfolded cytosolic state to a stable α-helical conformation [19]. *In vivo*, membrane association and diverse effects on lipid metabolism appear to be important components of the physiological function of α-syn in the CNS. It follows that disruption of lipid metabolism and lipid-membrane composition and integrity could play an important role in the pathogenesis of α-synucleinopathies. However, the specific role of oligodendrocyte membrane transport and lipid metabolism in MSA remains to be clearly elucidated.

The purpose of this review is to explore the significance of brain lipid metabolism in MSA pathogenesis. We will firstly discuss oligodendrocyte-specific processes, specifically myelin formation, which may underlie the unique glial focus of MSA pathology. The relevance of lipid membranes and metabolism to the normal physiology and dysfunction of α-syn will then be explored. Finally, epidemiological evidence linking membrane transport and lipid dyshomeostasis to MSA will be discussed.

## Review

### Oligodendrocyte-specific pathology

In an effort to distinguish processes specific to MSA pathogenesis, the physiology and maintenance of myelin presents a natural focus, given the primary lesion in MSA occurs within oligodendrocytes. Myelin in cross section is a spiral structure constituted by multiple layers of a specialized extension of the oligodendrocyte plasma membrane that wraps around axons (reviewed in [24]). The composition of myelin is characterized by high proportions of cholesterol and glycosphingolipids, which are arranged in a highly ordered structure with associated structural and signaling proteins. Hence, this highlights the importance in maintaining appropriate lipid concentrations, as they are crucial for the proper formation of myelin.

Myelin is traditionally known to function as an electrical insulator for axons to facilitate the rapid propagation of action potentials. However, Ravera and colleagues [25] have recently demonstrated that similar to mitochondria, isolated myelin vesicles possess the capacity to undergo oxidative phosphorylation to generate ATP for axons. Hence, myelin may hold additional roles that contribute towards axonal survivability. Therefore, defects in myelin arising from lipid dyshomeostasis could potentially perturb the survivability of axons to in turn cause neurodegeneration as a contributor towards MSA pathogenesis. The mechanism by which axonal degeneration induces neurodegeneration is discussed in a review by Wang et al. [26], although another recent study reported an absence of axonal degeneration following chronic dysmyelination in the Long-Evans shaker rat [27]. These rats are characterized by myelination abnormalities resulting from a mutation in the gene that encodes myelin basic protein [27]. While such conflicting findings are possibly the result of varying experimental conditions, clarification of the relationship between myelin loss, axonal degeneration and neurodegeneration is required.

Despite the conflicting findings on the link between myelin integrity, axonal survivability and neurodegeneration, the disruption of myelin has been proposed to be an early process in the pathogenesis of MSA by Song and colleagues [28]. More specifically, the authors observed abnormal subcellular relocalization of myelin-associated proteins, which appeared to precede α-syn aggregation into GCIs. The earliest changes observed included the relocalization of p25α from the myelin sheath to the cell body of oligodendrocytes, which was presumed to induce abnormal expansion of the cell body. Moreover, similar relocalization patterns were observed with myelin basic protein whilst also demonstrating increased degradation and accumulation in the expanded soma of affected oligodendrocytes. A previous study of myelin changes in MSA also noted that proteolysis of myelin basic protein was an early marker of oligodendrocyte pathology in affected brain areas and sometimes preceded the appearance of GCIs [29]. Substantiating the relevance of these observations to later α-syn aggregation in MSA, p25α co-localizes with α-syn in GCIs and is a potent inducer of α-syn aggregation *in vitro*[30-32]. Thus a disruption of myelin synthesis and maintenance in oligodendrocytes could represent the primary disturbance in MSA. A possible source of this disturbance could lie in the synthesis and handling of key myelin lipids by oligodendrocytes. The consequent alteration in myelin membrane composition would then be likely to impact upon membrane association with α-syn as a precedent to α-syn aggregation. In addition, with its putative role in diverse lipid biosynthetic pathways, α-syn expression may be governed by feedback mechanisms operating in response to myelin lipid abnormalities.

### α-Synuclein pathology

#### α-Synuclein oligomers are key cytotoxic species in α-synucleinopathies

The α-syn protein contains a highly hydrophobic 12–amino acid domain at its center (71-VTGVTAVAQKTV-82), which appears to be essential for fibrilization of the protein [33]. Deletion or disruption of this domain through the addition of a charged amino acid abrogates the ability of α-syn to form amyloid fibrils [33]. Insoluble aggregates of α-syn with this 12 amino acid stretch at their core [33] are the pathological hallmarks of PD/DLB and MSA in neurons and oligodendrocytes respectively. However, a significant body of evidence suggests that mature α-syn fibrils are not required for cytotoxicity in these disease processes. Instead, early misfolded forms of α-syn, possibly dimers or small oligomers, are proposed to be the primary toxic species [34]. Consistent with this hypothesis, investigations of α-syn mutants linked to familial forms of PD (A30P and A53T) have found that both forms undergo self-association to form soluble oligomers more rapidly than wild-type protein, however only A53T demonstrates faster formation of fibrils [35,36]. Underlying their toxicity, α-syn oligomers assume annular pore-forming configurations which may be capable of causing inappropriate permeabilization of cellular membranes [37]. Subsequent aggregation into visible fibrils may even serve as protective sequestration of this toxic species [34,38].

#### Formation of α-synuclein oligomers at the lipid membrane surface

As highlighted above, under normal conditions α-syn exists as randomly structured and natively unfolded monomer within the cytoplasm. However, at the membrane surface in the presence of lipids α-syn adopts a dramatic change in structure to a folded α-helical secondary structure [39]. A number of studies have suggested that the helix-rich membrane bound form of α-syn plays a crucial role in initiating the pathological aggregation of the protein. Accelerated α-syn aggregation has been observed as a result of α-syn exposure to long chain polyunsaturated fatty acids [40] and with increased binding to membranes and lipid droplets within cells [41]. In a study of isolated brain fractionates of α-syn, Lee and colleagues [42] observed progressive aggregation of the protein in the membrane fraction but no aggregation in the cytosolic fraction after 3 days. Moreover, addition of cytosolic α-syn to the membrane fraction accelerated aggregation, suggesting that initial self-association at the membrane may seed accumulation of the more abundant cytosolic form of α-syn.

#### Lipid membrane composition influences α-synuclein association

Additional studies of lipid membrane composition and structure shed further light on the mechanics of α-syn membrane association and dysfunction. Fortin and colleagues [18] observed that α-syn binds preferentially to lipid raft microdomains in biological membranes. Lipid raft microdomains have been shown to be essential for oligodendrocyte survival signaling by providing a favorable environment for growth factor-mediated integrin activation [43]. Lipid rafts are highly dynamic sterol- and sphingolipid-enriched structures, which compartmentalize many cellular processes occurring in biological membranes [44,45]. Quantitative manipulation of constituent lipid species leads to disorganization of lipid raft microdomains and dissociation of proteins bound to the lipid rafts [46,47]. Fortin and colleagues [18] noted that cholesterol extraction disrupted lipid rafts and dramatically reduced α-syn association *in vitro*. Conversely, Cole and colleagues [41] loaded HeLa cells expressing wild-type α-syn with free fatty acid and found significant increases in α-syn association with membranes and triglyceride-rich lipid droplets. Thus it appears that the dynamics of intracellular α-syn association are highly responsive to manipulation of lipid homeostasis and membrane composition. A number of studies have shown that the A30P α-syn mutant associated with familial PD displays defective binding to lipid membranes, while the A53T mutant shows no such difference compared to wild-type protein [18,48,49]. Although there are no known causative α-syn mutations in MSA, variants of the SNCA gene have been identified to be associated with an increased risk for MSA [11,50].

#### α-Synuclein plays a role in lipid-mediated signaling and synaptic vesicle function

α-Syn is expressed primarily by neurons, where it is especially enriched at their presynaptic terminals [18,51,52]. α-Syn is described as a natively unfolded protein since it forms a disordered ‘random coil’ configuration in its cytosolic state [53]. The association of α-syn with lipid membranes is well established by *in vitro* methods [19,39]. Lipid association, moreover, induces folding of the protein to a stable α-helical conformation [19,39,54]. However, the significance of these observations for the physiological function of the protein *in vivo* remains incompletely understood.

A study of α-syn structure found high homology of sequences at the C and N terminals to characteristic motifs of the fatty acid binding protein (FABP) family, suggesting α-syn may play a role in intracellular fatty acid transport [55]. However, subsequent findings have suggested a more nuanced relationship to lipid membranes and cellular lipid metabolism. The propensity of α-syn to bind and stabilize small lipid vesicles [19,41] and the subcellular localization of the protein at the presynaptic terminal in neurons [18,51,52] are suggestive of a role in synaptic transmission. Indeed, transgenic mice lacking α-syn (α-syn^-/-^) show deficits in nigrostriatal dopaminergic neurotransmission [20,56]. On a sub-cellular level, Cabin and colleagues [56] found that α-syn^-/-^ mice have a 50% reduction in size of undocked synaptic vesicle pool and synaptic vesicle depletion after high-frequency stimulation.

The mechanism of this influence on vesicle function and turnover may rely on the interaction of α-syn with phospholipase enzymes and associated lipid-mediated signal transduction. α-Syn binds to and inhibits the activity of phospholipase D (PLD) [57,58] – an enzyme involved in lipid-mediated signaling cascades responsible for regulating functions including cytoskeletal rearrangement and endocytosis [59]. PLD inhibition appears to be required for synaptic vesicle fusion with the target membrane and may regulate synaptic vesicle recycling. This is consistent with the results of Cabin and colleagues [56] who showed a reduction in the synaptic vesicle pool in α-syn^-/-^ mice. A further role in intracellular signaling was suggested by Narayanan and colleagues [60], who found that α-syn also binds to phospholipase Cβ (PLCβ), which is a G-protein coupled enzyme in the dopamine-signaling pathway that exerts downstream effects through calcium signaling by hydrolyzing phosphatidylionositol. This binding interaction was found to inhibit the catalytic activity of PLCβ by 50%. Later studies have suggested α-syn also prevents the degradation of a specific type of PLCβ – PLCβ1, to in turn promote calcium signaling [61]. Interestingly, A53T mutations in α-syn that are linked to familial PD are also associated with weaker binding to PLCβ [60]. Hence, this could potentially serve as a mechanism by which α-syn perturbs downstream effects of PLCβ as a consequence of reduced activation of lipid signaling pathways.

#### α-Synuclein exerts a wider influence on lipid metabolism in the brain

The *in vitro* findings summarized above are largely specific for neuronal physiology, which is characteristic of the bulk of α-syn research. However, the primary oligodendrocyte pathology exhibited in MSA demands a broader consideration of the function of this protein in the brain. A research team headed by E.J. Murphy of the Ohio State University has published a number of important studies suggesting a wider role for α-syn in brain lipid metabolism. This includes a study demonstrating that astrocytes cultured from α-syn gene-ablated mice have significantly disrupted uptake of the fatty acids palmitate and arachidonic acid with increased trafficking to the neutral lipid pool and decreased trafficking to phospholipids [62]. Again in whole brains of α-syn^-/-^ mice, uptake of palmitic acid was reduced by 35% and there was significantly altered incorporation into a number of phospholipid classes [63]. It is proposed that these effects on fatty acid uptake and trafficking are independent of any function as a FABP on account of the failure to demonstrate any binding of α-syn to oleic or palmitic acid using titration microalorimetry [63]. The mechanism may instead relate to α-syn regulation of acyl-CoA synthase activity, which mediates incorporation of palmitate into the acyl-CoA pool from where it is incorporated into individual phospholipids [63,64].

From the same research team, Ellis and colleagues [65] found a significant reduction in the linked complex I/III activity of the electron transport chain in mitochondria of α-syn^-/-^ mice. This was apparently mediated by reduced levels and altered fatty acid composition of cardiolipin, an important component of the mitochondrial membrane required for assembly and function of the electron transport chain. Of significance and unlike PD, there is no significant deficit in mitochondrial respiratory chain activity in MSA [66]. However, this may still be of particular relevance to MSA since oxidative stress precipitates MSA neuropathology, as evidenced by the potentiating effects of mitochondrial toxin 3-nitroproprionic acid on clinical deficits and neurodegeneration in transgenic mouse models of the disease [67,68]. While speculative, in MSA dysfunction of myelin respiratory chain activity may be affected due to alterations in myelin lipid homeostasis.

In summary, α-syn appears to have diverse roles in brain lipid metabolism, including lipid-mediated signaling pathways and fatty acid trafficking to key phospholipid pools integral to the structure and function of biological membranes. These functions may be integral to an understanding of the reciprocal influences of lipid membrane composition on pathological α-syn aggregation as discussed below.

### Pathophysiology mechanisms of lipids in MSA

#### Shifts in membrane-associated α-synuclein observed in MSA brains

The *in vitro* evidence for the pathogenicity of membrane bound forms of α-syn has been substantiated by several studies of human brain tissue fractionates from individuals affected by MSA and other α-synucleinopathies. Case–control analyses of α-syn solubility in human brain tissue have shown increases in sodium dodecyl sulfate (SDS)-soluble ‘membrane-associated’ α-syn in disease affected regions of MSA brains with concomitant decreases or no change in the buffer-soluble cytosolic fraction [69-74]. These observations lend support to the hypothesis that a solubility shift from the cytosolic to membrane compartment may be a fundamental process in α-syn pathology in α-synucleinopathies. Furthermore, one detailed comparative study of PD and MSA brain tissue [74] revealed distinct patterns of α-syn solubility between the disease groups. Along with the expected wider regional involvement in MSA, there was also much greater quantitative accumulation of membrane-associated α-syn in the MSA substantia nigra compared to PD. The extent of membrane associated α-syn accumulation also appeared to positively correlate with neurodegeneration in MSA (especially in the striatum) but not in PD. Hence, these disparities highlight that unique pathophysiologies of α-syn dysfunction are likely to operate in MSA and PD, which would explain the fundamentally different cellular focus of pathology in each disease.

#### Association of *COQ2* with pathogenesis of MSA

A recent collaborative study that combined linkage analysis with whole-genome sequencing of a post-mortem sample from a member of a multiplex family with a history of MSA, has provided the first piece of evidence linking *COQ2* to MSA [10]. More specifically, the common V343A variant in the *COQ2* gene was found to be associated with an increased risk of sporadic MSA in Japanese populations [10]. The *COQ2* gene encodes a biosynthetic enzyme in the production of coenzyme Q_10_ (also known as ubiquinone) [75]. Coenzyme Q_10_ is a lipid-soluble compound that acts as a powerful antioxidant and the main electron acceptor in the electron transport respiratory chain. It is present in membranes of all cells and tissues, and is vital for the intracellular transport of electrons from complex I and II to complex III in the respiratory chain [76]. It is of interest that the α-syn^-/-^ mice have dysfunction at this site in the respiratory chain [65]. While generally concentrated within mitochondria, myelin also possesses the capacity to undergo oxidative phosphorylation and generate ATP for axons through functional electron transport chains within myelin membranes [25], and therefore requires coenzyme Q_10_ to perform this function. By measuring the effects of the V343A variant in lymphoblastoid lines, the collaborators demonstrated the variant induced functional impairments in *COQ2*, which is consistent with the decreased coenzyme Q_10_ levels in MSA brains in comparison to control [10].

Dysfunction and decreased levels of coenzyme Q_10_ promotes oxidative stress through an inability to remove free radicals, ultimately facilitating apoptosis-induced cell death [77,78]. Thus in the context of MSA, dysfunctional and apoptotic oligodendrocytes as a consequence of altered coenzyme Q_10_ function could contribute towards an inability to form and maintain myelin, which may impact on the long-term viability of demyelinated axons (see above). Interestingly, deficiencies of coenzyme Q_10_ appear to manifest with some symptomatic features characteristic of MSA, such as cerebellar ataxia and atrophy [79,80].

According to Fünfschilling and colleagues [81] however, the disruption of oxidative phosphorylation in cultured oligodendrocytes by inhibiting the formation of the mitochondrial complex IV does not impede the ability of oligodendrocytes to myelinate axons. Instead, the authors proposed that oligodendrocytes are capable of compensatory glycolytic activity upon disruption of oxidative phosphorylation, as evidenced by a significantly increased glycolytic rate in affected oligodendrocytes. Therefore, the role of coenzyme Q_10_ in MSA is unclear and will require further in-depth investigation, although its role in contributing towards oxidative stress remains plausible (discussed below).

#### ABCA8 – a novel oligodendrocyte lipid transporter

**Figure 2:**
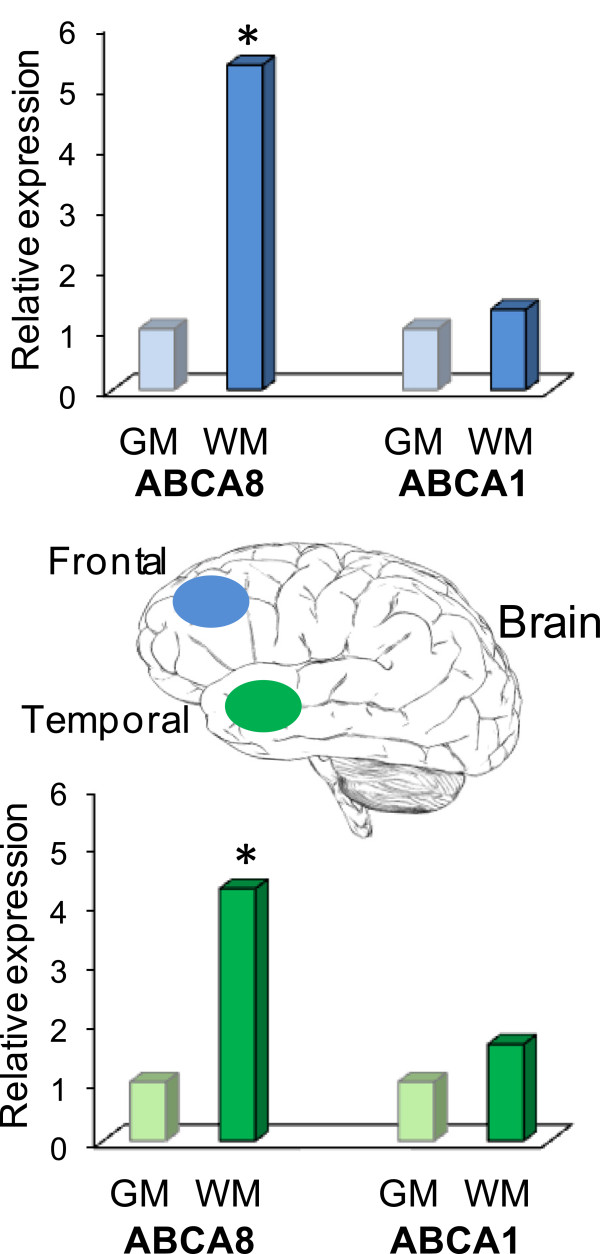
**Elevated expression of the lipid transporter ABCA8 in white matter regions of human brain.** The expression of ABCA8 and ABCA1 (prototype in A subfamily) was measured in the superior frontal grey matter (GM) and white matter (WM) and inferior temporal GM and WM from six normal adults, comprising of three males and three females, by quantitative PCR; *p < 0.01 (Student’s *t* test).

Besides *COQ2*, recent investigation of a novel member of the ATP-binding cassette (ABC) transporter family, ABCA8, has opened the way to a more detailed understanding of myelin formation and pathology. ABC transporters are transmembrane proteins that utilize the energy of adenosine triphosphate (ATP) hydrolysis to carry out certain biological processes [82,83]. The A subfamily of ABC transporters shares a common substrate specificity for lipid species [84] and several members have well characterized associations with human disease including neurodegeneration [85]. ABCA8 has recently been shown to be differentially expressed in multiple regions of adult human brains with significantly higher expression in oligodendrocyte-enriched white matter regions compared to grey matter cortical regions [86]. The expression of ABCA1 (the prototype in A subfamily) was unaltered in white matter regions, suggesting that ABCA8 may have a unique role in the brain white matter (Figure 2). Furthermore, in the same study, quantitative expression analysis in the prefrontal cortex across the human life-span showed increases correlating with age-associated myelination [87,88] and in conjunction with upregulation of the myelinating gene p25α. *In vitro*, ABCA8 was able to significantly stimulate both sphingomyelin synthase 1 expression and sphingomyelin production in a human oligodendrocyte cell line. Sphingomyelin is an important glycosphingolipid component of the myelin membrane. In sum these results strongly suggest that ABCA8 regulates lipid metabolism in oligodendrocytes and potentially plays a role in myelin formation and maintenance. It is likely that this ATP requiring transporter co-locates with respiratory chain proteins producing ATP within the myelin membrane to perform these roles.

The influence of ABCA8 on sphingomyelin metabolism may have cellular impacts beyond myelin structural integrity. Sphingomyelin is enriched in lipid-raft microdomains where it is thought to be an important mediator of membrane rigidity and permeability [89] and of compartmentalization of raft-associated cellular processes [90]. Sphingomyelin is also part of an important signaling pathway in which it is cleaved by sphingomyelinase to form ceramide which serves as a second messenger for cellular functions including cell cycle progression, proliferation, differentiation and apoptosis (reviewed in [91]). Sphingomyelin accumulation with or without significant sphingomyelinase deficiency are features of Niemann-Pick disease, of which subtypes A, C and D manifest with irreversible neurological damage [92].

Bleasel and colleagues [93] have tested the hypothesis that ABCA8 expression is associated with the disease process of MSA through involvement in the early disruption of myelin processing with subsequent impacts on the function and homeostasis of the key disease proteins α-syn and p25α. ABCA8 mRNA expression was significantly increased in MSA brains compared to controls in disease-affected grey matter (putamen and cerebellum) and disease-affected white matter underlying the motor cortex with no significant change in an unaffected region (visual cortex). ABCA8 and p25α expression were also positively correlated in disease-affected regions in both MSA and control tissue. These results strongly suggest that the upregulation of ABCA8 is associated at some level with the MSA disease process, possibly in response to reduced ATP production in myelin if *COQ2* is involved (discussed above). The positive correlation between ABCA8 and p25α may substantiate a link between ABCA8 and pre-GCIs disease events in line with the findings of Song and colleagues [28] outlined above. The relocalisation of p25α from the myelin sheath to the cell soma observed in this study was proposed to be part of a wider initial myelin dysfunction in MSA, consistent with a concurrent disruption of the putative myelin lipid transporter ABCA8.

The role of transcriptional upregulation of α-syn and other GCI proteins in the pathogenesis of MSA has been a contentious issue. Previous quantitative studies of MSA post-mortem tissue have failed to demonstrate α-syn overexpression at the mRNA level [15,94-97]. However it remain possible that small expression changes in oligodendrocytes could be masked by secondary neuronal death and dysfunction and more cell-specific expression studies are necessary. Other commentators have suggested that negative feedback mechanisms may secondarily suppress *SNCA* overexpression in established disease [95]. In an *in vitro* follow-up to the human tissue analysis cited above [93], overexpression of ABCA8 in cultured MO3.13 oligodendrocytes caused significant increases in expression of α-syn and p25α at the mRNA level. While caution is necessary attributing any causative role to ABCA8 in MSA pathogenesis at this stage, these results lend strong support to the relevance of myelin lipid dysregulation to α-syn pathology in oligodendrocytes.

### Association of cholesterol (rate-limiting lipid for myelination) and MSA

#### Higher serum cholesterol associated with decreased risk of MSA

In contrast to the numerous investigations of PD cohorts, only two case–control studies have examined the link between serum lipids and MSA. In the first study, Lee and colleagues [98] recruited 142 subjects with probable MSA and 155 age- and gender- matched controls for a cross-sectional analysis. The risk of MSA in the lowest quartile of total cholesterol (TC), low density lipoprotein (LDL) cholesterol and high density lipoprotein (HDL) cholesterol was significantly higher compared to the highest quartile of each. The ORs remained significant for low TC and HDL when adjusting for age, gender, use of cholesterol-lowering drugs, and histories of hypertension, diabetes mellitus, and smoking. The authors considered potential confounders including relative malnutrition with increasing severity of disease and use of a feeding tube, however these factors were not significantly correlated with lipid profiles. The recent second study of MSA in a Chinese population has confirmed the conclusion of the first study [99]. Other evidence linking particular dietary factors to MSA is conflicting however – meat and poultry consumption has been found to have both a positive [100] and negative [13] association to the disease in separate cross-sectional samples.

#### Correlation to PD evidence

The suggestion of a link between increased MSA risk and low serum cholesterol is consistent with some observational studies of PD, but the literature is marked by conflicting findings. Two prospective cohort studies have reported an inverse relationship between serum cholesterol and PD risk. de Lau and colleagues [101] reported a decreased risk of PD with increasing total cholesterol and evidence of a dose response relationship, however the association was restricted to women. Using self-reported total cholesterol levels, Simon and colleagues [102] also reported a modest decrease in relative risk with higher total cholesterol levels. Retrospective case–control studies have also reported a higher PD occurrence with lower TC and triglycerides [103] and with low LDL cholesterol [104]. In conflicting results, one prospective study [105] found an association between high total cholesterol at baseline and increased incidence of PD while another found no association [106]. Similarly, observational studies of dietary fat intake have reported a mix of conflicting associations: increased risk with increased total fat (particularly animal fat) [107-109], decreased risk with increased total fat and poly- and mono-unsaturated fatty acids (FA) [110]; increased risk with lower dietary cholesterol [111] and no association [112,113]. However, in a recent meta-analysis, Gudala and colleagues [114] revealed that there were no association between serum cholesterol and risk of PD, suggesting differences in lipid pathophysiology underlying PD and MSA.

#### Relevance of peripheral cholesterol homeostasis to the CNS

The mechanism for an increased risk of MSA with low levels of serum cholesterol is not obvious. The brain is the most cholesterol rich organ in the body with more cholesterol in the white matter due to myelin’s increased membrane density. Disturbances in cholesterol homeostasis may disrupt cell membranes where α-syn appears to exert some of its functions [101,105]. However, the fact remains that cholesterol-bearing lipoproteins in the peripheral circulation do not cross the blood brain barrier and most brain cholesterol is synthesized *in situ*[115]. On the contrary, oxidized cholesterol derivatives can easily diffuse across the blood brain barrier and may provide a mechanism of interaction between central and peripheral cholesterol pools. These derivatives include 24S-hydroxycholesterol, which appears to be involved chiefly in excretion of excess brain-synthesized cholesterol to the periphery [116] while 27-hydroxycholesterol demonstrates a net flux in the opposite direction where it concentrates in the white matter [117]. In further support of this possibility, Bosco and colleagues [118] reported increased levels of locally generated oxidative cholesterol metabolites in the cortices of DLB patients compared to controls, and a pro-aggregating effect of oxysterols on α-syn *in vitro*. The potential influence of peripheral cholesterol dyshomeostasis via 27-hydroxycholesterol is not implicated in the discussion of these findings. Thus the interaction of peripheral and CNS cholesterol homeostasis and its relevance to α-syn pathophysiology *in vivo* remain speculative.

Alternatively, coenzyme Q_10_ has been proposed to be a neuroprotective factor in PD pathogenesis due to its antioxidant properties [119,120]. Thus, since serum cholesterol is the strongest determinant of coenzyme Q_10_ serum levels [121], this could potentially explain reports of an inverse relationship between serum lipoprotein levels and PD risk [110]. Since lower serum cholesterol levels are associated with a decreased risk of MSA [98,99] and serum cholesterol determines coenzyme Q_10_ levels [121] it could be inferred that altered cholesterol homeostasis in turn alters the amount of coenzyme Q_10_ present. As deficiencies in coenzyme Q_10_ are known to induce oxidative stress [77,78], this then poses another mechanism by which peripheral lipid dyshomeostasis could contribute to the pathogenesis of MSA. This possibility is also in accordance with the finding that *COQ2* mutations were more common in MSA patients with predominant cerebellar involvement [10], as a previous study has shown the cerebellum in both rats and humans contains the lowest concentration of coenzyme Q_10_ in the brain [122]. Hence, it appears the cerebellum may have heightened vulnerability to the damaging consequences of mutations in the *COQ2* gene.

## Conclusions

In conclusion α-syn is a predominantly neuronal protein, which associates with lipid membranes and plays a role in synaptic vesicle function and turnover. In addition α-syn has wider roles in brain lipid metabolism, which are likely to be important to both neuronal and glial cell function. The studies summarized above provide a guide for the interaction of lipid dyshomeostasis, altered membrane composition and α-syn dysfunction. Oligodendrocytes are responsible for organising and maintaining the bulk of brain lipid in the form of the specialised myelin membrane. Myelin instability, potentially mediated by abnormalities of ABCA8 lipid transporter expression, may be an important precursor to α-syn pathology in MSA, consistent with the unique oligodendrocyte focus of the disease. ABC transporters require ATP and functional mutations in *COQ2* affecting coenzyme Q_10_ affect respiratory chain production of ATP. Together these deficits may contribute to the death and dysfunction of oligodendrocytes in MSA. Future studies should give due focus to the unique lipid-related processes underlying MSA pathology in contrast to the neuronal α-synucleinopathies, especially energy production required for sphingolipid processing and myelin membrane integrity.

## Competing interests

The authors declare that they have no competing interest.

## Authors’ contributions

WSK conceived and structured the review idea. JMB, JHW and WSK carried out the literature search and wrote the manuscript. WSK and GMH critically read and revised the manuscript. All authors read and approved the final manuscript.
